# High Dose C6 Ceramide-Induced Response in Embryonic Hippocampal Cells

**DOI:** 10.3390/biom15030430

**Published:** 2025-03-17

**Authors:** Federico Fiorani, Martina Mandarano, Samuela Cataldi, Alessandra Mirarchi, Stefano Bruscoli, Francesco Ragonese, Bernard Fioretti, Toshihide Kobayashi, Nario Tomishige, Tommaso Beccari, Claudia Floridi, Cataldo Arcuri, Elisabetta Albi

**Affiliations:** 1 Department of Pharmaceutical Sciences, University of Perugia, 06123 Perugia, Italy; federico.fiorani@dottorandi.unipg.it (F.F.); samuela.cataldi@gmail.com (S.C.); beccari.tommaso@unipg.it (T.B.); 2 Department of Medicine and Surgery, University of Perugia, 06126 Perugia, Italy; martina.mandarano@unipg.it (M.M.); alessandra.mirarchi@dottorandi.unipg.it (A.M.); stefano.bruscoli@unipg.it (S.B.); claudia.floridi@unipg.it (C.F.); cataldo.arcuri@unipg.it (C.A.); 3 Department of Chemistry, Biology and Biotechnologies, University of Perugia, 06123 Perugia, Italy; francesco.ragonese@unipg.it (F.R.); bernard.fioretti@unipg.it (B.F.); 4 UMR 7021 CNRS, Université de Strasbourg, 67401 Illkirch, France; toshihide.kobayashi@unistra.fr (T.K.); nario.tomishige@unistra.fr (N.T.); 5 Cellular Informatics Laboratory, RIKEN, Wako 351-0198, Saitama, Japan

**Keywords:** C6 ceramide, embryonic hippocampal cells, caspase 3, MIB, sphigomyelin, sphingomyelinase, sphingosine 1 kinase 2

## Abstract

Ceramide is a critical molecule in both the physiology and pathology of the central nervous system. The most studied aspect is its effect on embryonic/stem cells. A salient question is whether low doses of ceramide induce neuronal differentiation without interfering with sphingolipid metabolism and whether high doses can be used in glioblastoma for their cytotoxic effect. Here, we examined the effect of a high dose of ceramide (13 µM) on HN9.10e cells. Interestingly, 13 µM ceramide induced an immediate increase in cell viability, followed by an increase in the number of mitochondria. Microscopic and morphometric analysis revealed a decrease in the number of differentiated cells with 13 µM compared to 0.1 µM but with longer neurites. Furthermore, the lipidomic study demonstrated an increase in the formation of medium–long-chain ceramide and sphingomyelin species and sphingosine 1 phosphate. Sphingolipid modification correlated with *SMPD3*, *ASAH2*, and *SPHK2* gene expression coding for neutral sphingomyenase 2, ceramidase 2, and sphingosine kinase 2, respectively. Overall, our data show that the variety of responses to ceramide of the same cell type is dependent on the concentration used. Low doses do not affect sphingolipid metabolism, and high doses do so with a different cellular response.

## 1. Introduction

Ceramides (Cers) are a class of bioactive molecules belonging to the large family of sphingolipids (SphL). Individual Cer species are grouped into Cers containing medium-chain fatty acids (C12–C14), long-chain fatty acids (C16–C18), very-long-chain fatty acids (C20–C24), and ultra-long-chain fatty acids (≥C26). Furthermore, Cer species can be classified as unsaturated, monounsaturated, or polyunsaturated. Cers are synthesized de novo in the endoplasmic reticulum from palmitic acid and serine, which are synthesized by a serine palmitoyltransferase to produce 3-ketosphinganine [1]. A 3-ketodihydrosphingosine reductase then induces the synthesis of sphinganine. Only at this point does a Cer synthase provide the addition of a fatty acid to produce dihydroceramide, which is reduced to ceramide by a desaturated dihydroceramide [1]. Once synthesized, Cers are translocated to the Golgi apparatus by a Cer transporter protein (CERT). Here, ceramide-1-phosphate (C1P), sphingomyelin (SM), or more complex lipids such as gluco- and galactocerebrosides and gangliosides can be formed [2]. In the opposite direction, Cers can also be derived from the degradation of gluco- and galactocerebrosides, gangliosides, and SM by sphingomyelinases, of which there are different isoforms: acid, neutral, and alkaline [3]. Whatever the origin, Cers can be degraded by ceramidases (Cerase) to sphingosine, and this can be phosphorylated to sphingosine-1-phosphate (S1P) thanks to sphingosine kinases (SPHK) [2]. In the laboratory, C6 Cer is extensively used because it is more efficient at passing through the plasma membrane than Cers with a longer acyl chain [3]. In cells, C6 Cer is used to form endogenous Cer. Of note, C6 Cer is incorporated into longer species of Cer via de novo synthesis [4]. Ogretmen et al. demonstrated that C6 Cer can generate endogenous long-chain Cer not via the elongation of its fatty acids but via deacylation/reacylation [5]. Cer acyl chain length and unsaturation influence membrane lipid raft function [6]. With the discovery that Cers are essential in membrane function, Cers have emerged as important bioactive molecules for membrane angles and, as a consequence, for the formation and release of exosomes [3]. Intracellular levels of Cers accumulate in response to a myriad of pro-apoptotic, pro-autophagy, pro-proliferation/differentiation, pro-immune-response, and pro-endoplasmic-reticulum-stress stimuli [1,3].

Several lines of evidence have indicated the implication of Cers in cancer cell apoptosis and chemotherapy sensitivity. First, Cer has powerful potential as a tumor suppressor since it induces cell death in several cancer cell lines and in vivo [7]. Of note, C6 Cer delays cutaneous T cell lymphoma cell viability and induces apoptosis [8]. Its antiproliferative and proapoptotic action makes C6 Cer active in canine mammary cancer [9]. Moreover, C6 Cer inhibits the motility of thyroid carcinoma cells, reducing the number of cells forming lamellipodia [10]. Second, 4T1 breast tumor cells are highly resistant to C6 Cer [11]. C6 Cer resistance was studied by changing the expression of genes for protein involved in Cer metabolism, membrane biology, and vesicular trafficking as CRISPR-Cas9 [12]. Third, C6 Cer is considered a potent chemotherapeutic drug for glioblastoma [13]. The dysregulation of Cer metabolism or low levels of Cers are implicated in cancer drug resistance [14].

Furthermore, C6 Cers also play a role in several other pathophysiology conditions: they inhibit SARS-CoV-2 viral replication [15]; change the degenerative cell viability of the nucleus pulposus and cell apoptosis [16]; induce the death of cardiomyocytes [17]; reduce the beneficial anti-inflammatory effects of Secukinumab administered to protect against the development of neurological deficits following hemorrhage [18]; and exert anti-inflammatory action by improving the lipid profile, the antioxidant system, and energy homeostasis in non-alcoholic steatohepatitis [19].

To obtain these effects, 6–25 µM C6 Cer concentrations were used. In our laboratory, it has been previously demonstrated that low doses of C6 Cer corresponding to 0.1 µM induce the differentiation of embryonic hippocampal cells. The release of exosomes rich in miRNAs that regulated the expression of genes for differentiation and their incorporation into neighboring cells were responsible for the diffusion of the differentiation signal [3].

It is now clear that high doses of C6 Cer play a role in several pathological conditions; however, the effect of high concentrations of C6 Cer has not been previously addressed in embryonic cells. Therefore, we aimed to investigate the potential role of high doses of C6 Cer in embryonic hippocampal cells.

## 2. Materials and Methods

### 2.1. Cell Culture and Treatments

HN9.10e (a kind gift from Kieran Breen, Ninewells Hospital, Dundee, UK) are immortalized hippocampal neurons, as previously reported [3]. They were grown in Dulbecco’s Modified Eagle Medium (DMEM) supplemented with 10% FBS, 2 mM l-glutamine, 100 IU/mL penicillin, 100 μg/mL streptomycin, and 2.5 μg/mL amphotericin B [3,20]. Cells were maintained at 37 °C in a saturating humidity atmosphere containing 95% air and 5% CO_2_. C6 Cer was added to the culture medium at 0.1 µM and 13 µM concentration.

### 2.2. Cell Viability

To test cellular viability, a 3-(4,5-dimethylthiazol-2-yl)-2,5-diphenyltetrazolium bromide (MTT) assay was used, as previously reported [20].

### 2.3. Flow Cytometry Analysis for Measurement of Cell Cycle and Apoptosis

Samples were treated under five conditions: untreated control, two concentrations of ceramide (0.1 µM and 13 µM), and H_2_O_2_ at 97.9 mM as a positive control for the apoptosis. After treatment, cells were collected and centrifuged at 300× *g* for 10 min to recover cells in suspension. The cells were washed twice with phosphate-buffered saline (PBS) and prepared for cell cycle analysis using propidium iodide (PI) staining. Cells were resuspended in 0.4 mL of a hypotonic fluorochrome solution containing 0.1% Triton X-100, 50 μg/mL propidium iodide, and 0.1% sodium citrate. The cell suspension was transferred to 12 × 75 mm polypropylene tubes (BD Biosciences Italy, Milano, Italy) and incubated for at least 30 min in the dark at 4 °C to ensure complete staining. Stained samples were then analyzed using a Coulter Epics XL-MCL Flow Cytometer (Beckman Coulter, California, USA) equipped with a 488 nm laser for the excitation of PI. The propidium iodide fluorescence of individual nuclei was measured, and data were collected for at least 10,000 events per sample. The percentages of cells in the G_0_/G_1_, S, and G_2_/M phases, as well as apoptotic cells (sub-G_1_ population), were calculated using FlowJo 10 software.

### 2.4. Mitochondrial Activity and Biogenesis

For the evaluation of membrane potential and mitochondria biogenesis, the Tetra Methyl Rhodamine Methyl ester (TMRM) stain (Sigma-Aldrich-Merk Life Science S.r.l., Milan, Italy) and Mitotracker Green FM (Invitrogen-Thermo Fisher, Monza, Italy) were used. Cells were incubated in DMEM with 30 nM of TMRM and 100 nM of Mitotracker Green for 20 min. After incubation, plates were washed twice with PBS and analyzed with an Axio Examiner florescence microscopy (Zeiss, Castiglione Olona, Varese, Italy) using a Rhodamine filter for TMRM and Fluorescein isothiocyanate Dye Filter (FITC) for Mitotracker Green according to the manufacturer’s instructions.

### 2.5. Cell Morphology

HN9.10e cells were cultured as reported above for 24, 48, 72, and 96 h in the absence or presence of 0.1 µM C6 Cer and 13 µM C6 Cer. The observations were performed by using the inverted microscope EUROMEX FE 2935 (ED Amhem, The Netherlands) equipped with a CMEX 5000 camera system (40× magnification).

### 2.6. Immunocytochemistry

HN9.10e were cultured for 24 h for immunocytochemical analysis, performed as previously reported [20].

### 2.7. Ultrafast Liquid Chromatography–Tandem Mass Spectrometry

Lipid extraction, the preparation of external or internal standards, and the analysis and identification of each species were performed as previously reported [21]. For standards, 12:0 SM, 16:0 SM, 18:1 SM, 24:0 SM, C18:0 sphinganine, C6:0 Cer, C16:0 Cer, C18:0 Cer, C20:0 Cer, C24:0 Cer, C16:0 glucosylceramide (GluCer), and sphingosine-1-phosphate (S1P) were used.

### 2.8. Fluorescence

An SM probe, enhanced green fluorescent protein-nontoxic-lysenin (EGFP-NT-Lys), was used to localize SM. The probe was purified from the *E.coli* strain BL21(DE3) harboring pET28/EGFP-NT-Lys according to Tomishige et al. [22], with slight modification [3].

### 2.9. Reverse Transcription Quantitative PCR (RTqPCR)

Total RNA was extracted from NH9.10e cells cultured for 24 h as previously described [3]. The following target genes were analyzed: *SM phosphodiesterase 3 (SMPD3*, Hs04187047_g1), *neutral ceramidase (ASAH2*, Hs01015655_m1), and *sphingosine kinase 2 (SPHK2*, Hs01016543_g1). *Glyceraldehyde-3-phosphate dehydrogenase (GAPDH*, Hs99999905_m1) and *18S rRNA (S18*, Hs99999901_s1) were used as housekeeping genes. mRNA relative expression levels were calculated as 2^−ΔΔ^*^C^*^t^ by comparing the results of VD3-treated sample with those of untreated samples.

### 2.10. Statistical Analysis

Data were expressed as means ± SD of three independent experiments performed in duplicate, and significance was verified by Student’s *t*-test.

## 3. Results

### 3.1. Ceramide Treatment Influences Cell Viability and Does Not Affect the Cell Cycle Progression and Apoptotic Levels of Cells

Previous results from our group implicated 0.1 µM Cer in the release of exosomes from HN6.10e cells spreading the differentiation signal in neighboring cells [3]. In the experimental plan, the Cer dosage had been chosen on the basis of vitamin D3-induced Cer production. To determine the effect of high doses of Cer, known to be involved in different pathologies and/or to have a therapeutic effect (see introduction), HN9.10e cells were first treated with C6 Cer using different doses, with the aim of understanding whether any applications of high doses could be harmful to embryonic cells.

**Figure 1:**
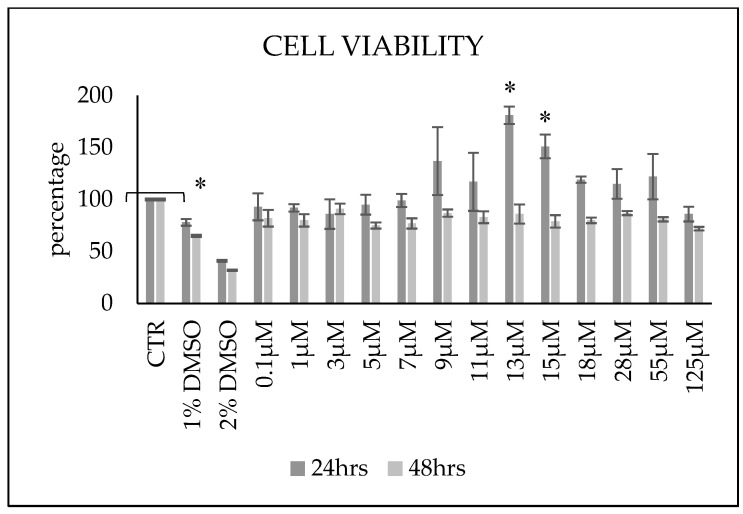
Effect of increasing doses of Cer on HN9.10e cell viability after 24 h and 48 h of treatment. DMSO was used as a positive control. Data are expressed as percentages with respect to the control sample (CTR) (100%) and represent the mean ± SD of 3 independent experiments performed in duplicate. DMSO treatment was used as a positive CTR. * *p* < 0.05 versus CTR.

After Cer treatment for 24 h, cells underwent a slight non-significant reduction in viability compared to 0.1 µM up to 7 µM then started recovering, with maximal effects seen with 13 µM. Since it has been shown that HN9.10e reaches the maximum DNA synthesis in the cell cycle at 24 h and then declines strongly at 30 and 36 h, it is expected that at 48 h, the second cell cycle will have restarted [20]. Therefore, the subsequent analysis was set at 48 h. After 48 h, cell viability was similar at all concentrations tested (Figure 1). So, we decided to use 0.1 µM as a low dose example and 13 µM as a high dose example.

To evaluate the biological effects of low and high concentrations of Cer, we next determined the cell cycle profile of cells after treatment with 0.1 µM and 13 µM Cer.

**Figure 2:**
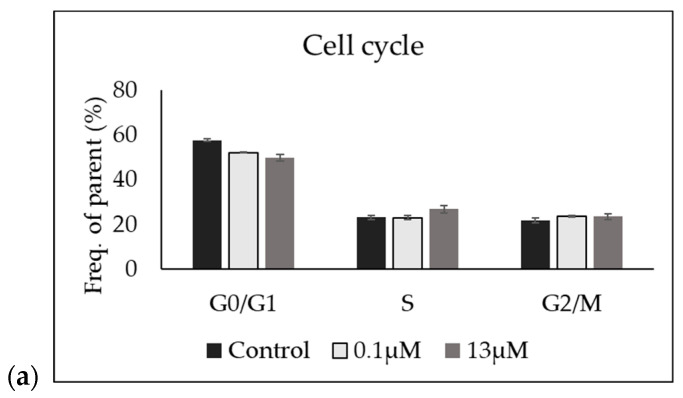

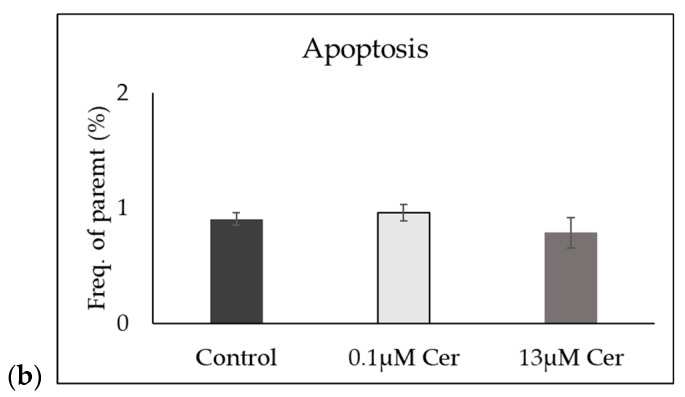
FACS analysis. Effect of 0.1 µM and 13 µM Cer on HN9.10e cell cycle (**a**) and apoptosis (**b**). The data represent the mean ± SD of 3 independent experiments performed in duplicate.

Thus, samples were treated for 48 h under four conditions: untreated control (CTR), two concentrations of Cer (0.1 µM and 13 µM), and H_2_O_2_ (97.9 mM) as a positive control for apoptosis. Following treatment, cells were stained with propidium iodide for DNA content analysis and analyzed using an FACS can flow cytometer. The percentages of cells in the G_0_/G_1_, S, and G_2_/M phases, as well as the apoptotic sub-G_1_ population, were calculated and compared across all treatment groups. No significant changes were observed in the cell cycle distribution or in the apoptotic sub-G_1_ population in any of the Cer-treated groups compared to the untreated CTR (Figure 2). The H_2_O_2_-treated positive control showed a slight increase in the sub-G_1_ population, but this difference was not statistically significant, confirming the assay’s capability to detect apoptotic changes.

Interestingly, these results indicated that treatment with Cer, at the concentrations tested, did not significantly alter cell cycle progression or induce apoptosis under the experimental conditions used.

**Figure 3:**
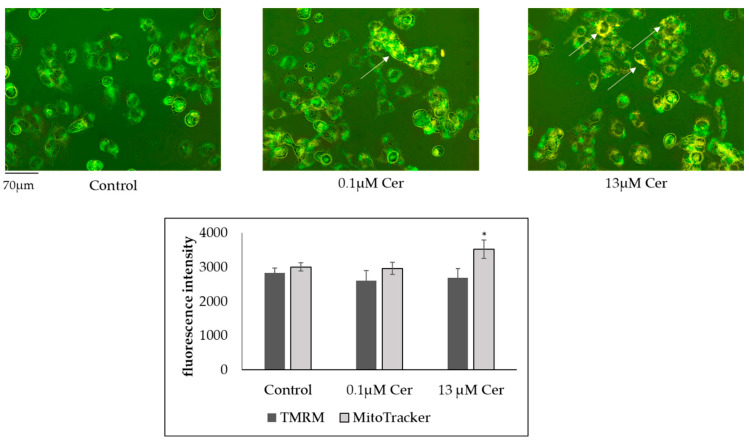
Merge images of the accumulation of TMRM (orange) and Mitotracker Green staining (green) of HN9.10e cell mitochondria in control conditions or treated with 0.1 μM Cer and 13 μM Cer. Arrows represent the co-localization. Fluorescence intensity of TMRM and Mitotracker Green measured in at least 30 cells both under control and treated conditions. * *p* < 0.05 of 0.1 μM Cer- and 13 μM Cer-treated samples versus the control sample.

Because the mitochondrion is a key integrator of apoptotic signals, to confirm the above result, the effects of 0.1 µM and 13 µM Cer on mitochondrial mass and function were assessed after 48 h of treatment. Thus, mitochondria were analyzed by measuring mitochondria membrane potential using the tetramethylrhodamine ethyl ester (TMRE) assay [23]. Mitochondrial mass and transmembrane potential were measured using MitoTracker Green and MitoTracker Orange dyes, respectively [24]. The results showed a significant increase in the values obtained with a MitoTracker test with 13 µM Cer treatment, where TMRM values did not vary. This may suggest an increase in the number of mitochondria in the treated cells compared to the control (Figure 3).

### 3.2. High Concentrations of Ceramide Increase the Length of Neurites

**Figure 4:**
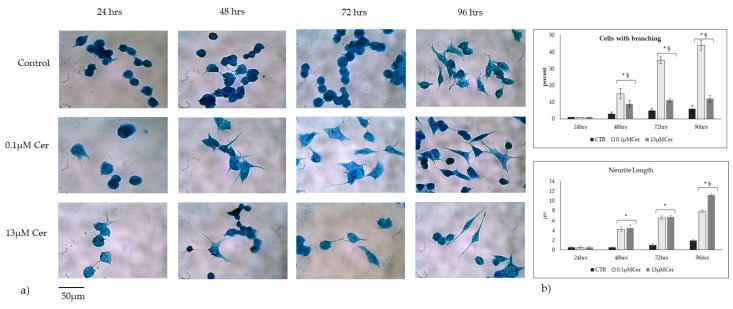
Effect of ceramide on HN9.10e cells (**a**,**b**). Morphology analysis after 24, 48, 72, and 96 h of cell culture. HN9.10e cells were cultured without (CTR) or with 0.1 µM and 13 µM Ceramide (Cer). The observations were performed by using the inverted microscope EUROMEX FE 2935 equipped with a CMEX 5000 camera system, with 20× magnification. The morphometric analysis was performed by using ImageFocus software, version 2.5. The data represent the mean ± SD of 3 independent experiments performed in duplicate. * *p* < 0.05 with 0.1 µM Cer and 13 µM Cer treatment versus the control (CTR) sample; ^§^ *p* < 0.05 with 13 µM Cer versus 0.1 µM Cer.

The role of 0.1 µM Cer in HN9.10e cell differentiation was reported previously by our group after 72 h and 96 h of treatment [3]. So, we performed a morphologic time course study with the low dose (0.1 µM) Cer and the high dose (13 µM) Cer. The number of seeded cells is identical in all samples (1 × 10^5^/mL). The difference in the number of cells framed/field depends on their way of growing together and/or differentiating and establishing connections. The results demonstrated that the number of cells undergoing differentiation with 13 µM Cer was higher than in the control and lower than with 0.1 µM Cer. However, after 96 h of treatment, the neurite length was greater with 13 µM Cer than with 0.1 µMCer (Figure 4). The morphologic differentiative effect is similar to that previously obtained with SM [20] or vitamin D3 [25] in the same cells.

**Figure 5:**
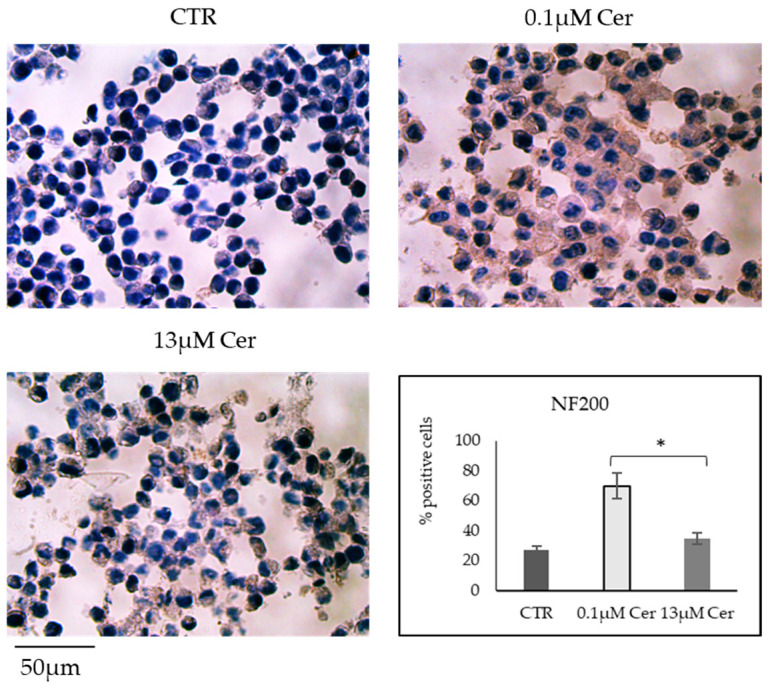
Immunocytochemical analysis of Neurofilament 200 kDa (NF200) expression in HN9.10e cells cultured in the absence or presence of 0.1 µM and 13 µM Ceramide (Cer). The observations were performed by using the inverted microscope EUROMEX FE 2935 equipped with a CMEX 5000 camera system, with 20× magnification. The data represent the mean ± SD of 3 independent experiments performed in duplicate. * *p* < 0.05 with 0.1 µM Cer and 13 µM Cer treatment versus the control (CTR) sample.

To confirm whether the number of cells undergoing differentiation was different in relation to the two concentrations of Cer used, the expression of the neurofilament protein (NF200) was evaluated after 24 h of treatment. The results showed a higher percentage of immunolabeled cells with both 0.1 µM Cer and 13 µM Cer compared to CTR. However, the percentage of cells expressing NF200 with 13 µM Cer was lower than in cells treated with 0.1 µM Cer, supporting the previous result (Figure 5).

### 3.3. High Level of C6:0 Cer Influences Sphingolipid Metabolism

**Figure 6:**
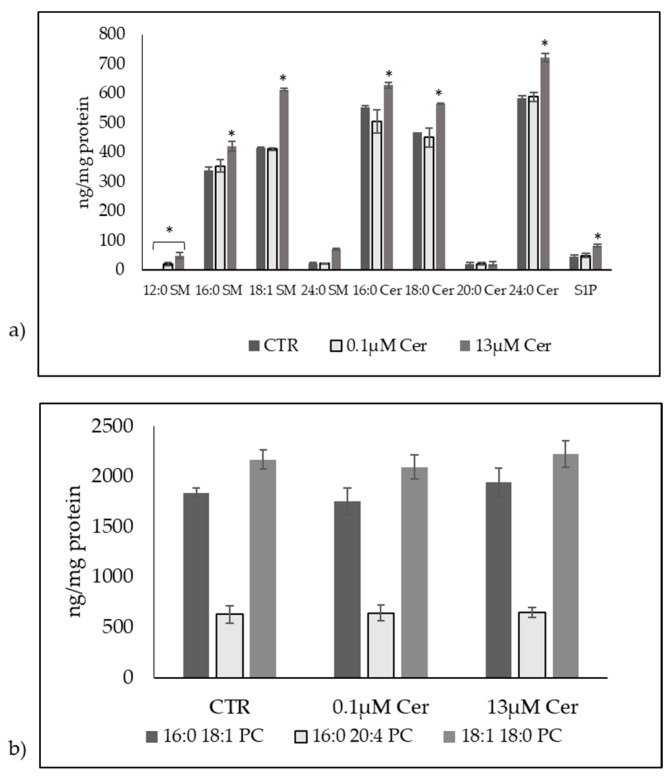
Mass spectrometric analysis after the treatment of HN9.10e cells with 0.1 µM ceramide (Cer) or 13 µM Cer. (**a**) Sphingomyelin (SM) and ceramide (Cer) profile; (**b**) phosphatidylcholine (PC) profile. The data represent the mean ± SD of 3 independent experiments performed in duplicate. * *p* < 0.05 with 0.1 µM Cer and 13 µM Cer treatment versus the control (CTR) sample.

Several studies have reported that C6 Cer, a Cer analog, has been extensively used in the laboratory due to its efficiency at passing through the plasma membrane [3]. Previously, we reported that C6 Cer at 0.1 µM dose did not influence the level of SM and Cer species in HN9.10e cells after 48 h of treatment. Therefore, it was of interest to examine the effect of 13 µM Cer treatment for 48 h on the cellular SM and Cer species. Thus, we analyzed SM and Cer species and S1P with lipidomic analysis by using external calibrators and internal standards as reported in the Section 2. The recovery of C8:0 Cer was 83%, 87%, and 85% in the control, 0.1 µM Cer-treated sample, and 13 µM Cer-treated sample, respectively. We analyzed the most common SM and Cer species by using external calibrators for each species, as reported in the Section 2. We have observed that 0.1 µM Cer did not induce significant changes in the 16:0 SM, 18:1 SM, 24:0 SM, 16:0 Cer, 18:0 Cer, 20:0 Cer, and 24:0 Cer species, supporting previous results [3]. We found that 12:0 Cer was below the detection limit in these experiments in the control and had a value of 21 ± 5 ng/mg protein after treatment with 0.1 µM Cer. The S1P value was 46 ± 6 ng/mg protein in the control sample, and it did not change significantly after the treatment of the cells with 0.1 µM Cer. After 13 µM Cer treatment, there were consistent increases in 16:0 SM (1.24-fold), 18:1 SM (1.47-fold), 24:0 SM (3.00-fold), 16:0 Cer (1.13-fold), 18:0 Cer (1.2-fold), and 24:0 Cer (1.43-fold) species; 12:0 Cer reached a value of 50 ± 11 ng/mg protein; and 20:0 Cer showed no significant changes. The level of S1P was also elevated by around 80%. Next, we analyzed phosphatidylcholine (PC) species for comparison, by using 16-0 18-1 PC, 16-0 20-4 PC and 18-1 18-0 PC external calibrators. All PC species showed no significant changes with either 0.1 µM Cer or 13 µM Cer (Figure 6).

**Figure 7:**
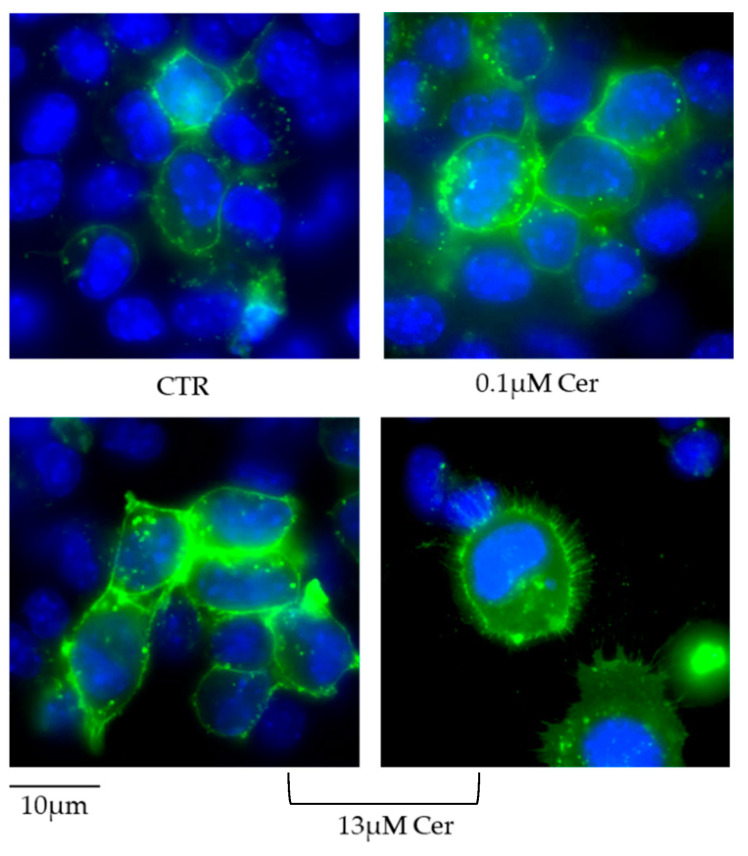
Presence of sphingomyelin in the control (CTR) sample, 0.1 µM ceramide (Cer) sample, and 13 µM Cer sample of HN9.10e cells with the EGFP-NT-Lys fluorescent probe.

The increase in SM content with 13 µM Cer in HN9.10e cells was confirmed by immunofluorescence using the EGFP-NT-Lys probe, as reported in the Section 2 (Figure 7).

**Figure 8:**
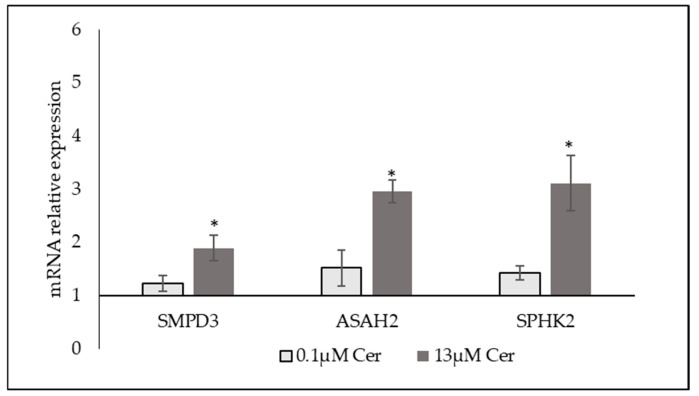
Effect of 0.1 µM ceramide (Cer) or 13 µM Cer on the gene expression of *SMPD3, ASAH2,* and *SPHK2*. *GAPDH* and *18S rRNA* were used as housekeeping genes. mRNA relative expression levels were calculated as 2^−ΔΔCt^, comparing the results of the treated samples with the control sample equal to 1, the origin of the axes. Data are expressed as the mean ± SD of 3 independent experiments performed in duplicate. * *p* < 0.05 versus CTR.

These results indicated that high level of exogenous C6 Cer might potentially regulate the endogenous sphingolipid metabolism in the cells. To better clarify this point, the gene expression of the *SMPD3* gene coding for neutral sphingomyelinase 2 (nSMase), the *ASAH2* gene coding for ceramidase 2 (Cerase2), and the *SPHK2* gene coding for the protein of the same name, sphingosine kinase 2 (SPHK2), was measured. The results showed a significant overexpression of all three genes analyzed only with 13 µM Cer (Figure 8).

## 4. Discussion

In this study, we examined the involvement of C6 Cer, implicated in mediating different pathophysiology processes including cancer, in embryonic hippocampal cell metabolic changes. Intriguingly, 13 µM Cer induced a strong increase in cell viability 24 h after treatment, which returned to values similar to those obtained with all other concentrations used in the study (from 0.1 µM to 125 µM) at 48 h. Data suggested a rapid metabolic response of the cells that did not occur at low and very high Cer concentrations, followed by cellular adaptation. It is currently very difficult to establish the exact reason for the increase in vitality at 24 h followed by a reduction at 48 h. Considering that high levels of Cer are correlated with low levels of calcium [26] and that low levels of calcium are correlated with high cell viability [27], it is possible to hypothesize that the administration of high levels of Cer may induce a modification to intracellular calcium levels, which disappear over time. Future studies could be designed to evaluate intracellular calcium after Cer treatment.

Recent studies have indicated 8 µM C6 Cer as an anticancer molecule in canine mammary cancer due to its antiproliferative and proapoptotic action [9]. It was found that 10 µM C6 Cer inhibited the motility of thyroid carcinoma cells [10] and 20 µM C6 Cer facilitated the metastasis of lung adenocarcinoma freeing extracellular vesicles [4], and 25 µM C6 Cer was suggested to be a potent chemotherapeutic since it induced cytotoxicity and death in glioblastoma cells [14] and T cell lymphoma cells [8]. Thus, different cell lines responded to different concentrations of Cer. In all the cited papers, C6 Cer was added to the culture medium without considering its endogenous level in the cells. We have previously reported that in HN9.10e cells, the level of C6 Cer was not detectable and the sum of the Cer species analyzed was equal to approximately 70 ng/mg protein [21], equal to approximately 0.14 µM/mg protein, considering an average molecular weight of all Cer species. Therefore, the dosage of 0.1 µM used was similar to the endogenous one related to proteins, and 13 µM was not a physiological dose.

In our experimental model, the 13 µM Cer concentration represented a particular dose used to obtain a rapid response in the physiological cells, which were embryonic hippocampal cells. At 48 h, when the cells had adapted to the treatment, no cytotoxic effect was demonstrated. In fact, cells in different phases of the cell cycle and cells undergoing apoptosis were comparable to the control cells and cells treated with 0.1 µM Cer. At the same time, in the absence of apoptosis, there was no change in mitochondrial activity, either. In contrast, the number of mitochondria was higher with 13 µM Cer treatment than the control and 0.1 µM Cer, although viability was established, indicating that intracellular changes persisted even at 48 h. Given that we observed a significant and rapid increase in cell viability and a later increase in the number of mitochondria, since there was a low level of Cer induced HN9.10e cell differentiation [3], we postulated a possible particular differentiation response in the cells over time. Surprisingly, the amount of cells undergoing differentiation was lower than that obtained with 0.1 µM Cer, as shown by the morphological and expression analysis of NF200, but the neurites that formed were longer. It is possible to hypothesize that Cer, if administered at high doses, is incorporated more easily into some cells than others.

It has been shown that the quantity and distribution of mitochondria in brain cells influence brain energy metabolism by regulating neuronal differentiation and neurite growth, as well as the formation of synapses and the release of neurotransmitters [28]. The possibility that the cell viability observed at 24 h, which in itself is linked to mitochondria, may be linked to the formation of well-differentiated cells with long neurites cannot be excluded. Future studies will be needed to investigate longer periods of mitochondrial function, i.e., by evaluating PGC-1alpha, mfn2, and opa1 mitochondrial biogenesis-related proteins. This is only a pilot study.

Additionally, 13 µM Cer exerted its biological effects primarily through the modulation of intracellular SM and Cer metabolism and the generation of the signaling molecule S1P. In HN9.10e cells, 0.1 µM Cer failed to influence sphingolipid metabolism at all, supporting previous findings [3]; 13 µM Cer increased the level of longer-chain Cer species. It is known that exogenous C6 Cer is used in the cells to form longer species of Cer via de novo synthesis [4]. Finding a higher number of medium–long-chain SM species in the cells suggested that the newly formed Cer was used as a substrate to synthesize SM. This increase was probably a stimulus to increase the expression of the gene encoding nSMase (*SMPD3*). Similarly, increased Cer could stimulate the expression of the gene encoding ceramidase (*ASAH2*) that degrades Cer to sphingosine. The latter could be the cause of the increased expression of the *SPHK2* gene by stimulating the synthesis of S1P, as demonstrated by our results. S1P is known to be critical in neurogenesis through effects on neural cell survival and growth [29]. It stimulated proliferation and morphological changes in neural progenitor cells [30], induced neuronal differentiation [31], and promoted the proliferation of retinal progenitors and their differentiation into photoreceptors [32]. Therefore, it is possible to hypothesize that the formation of neurites in HN9.10e after treatment with 13 µM Cer involves the metabolism of sphingolipids, unlike what happens with 0.1 µM Cer, which probably induces differentiation via another route.

## 5. Conclusions

Overall, our data show that the variety of responses to Cer of the same cell type is dependent on the concentration used. High doses of 13 µM Cer, also used for brain tumor pathologies, do not induce cytotoxicity in embryonic hippocampal cells. Although the dose is high, Cer induces differentiation in a lower number of cells but with the formation of longer neurites compared to the 0.1 µM dose, probably via S1P.

## Data Availability

All data are in the paper.
